# Profile of Elderly Patients Hospitalized for the First Time in Psychiatry

**DOI:** 10.1192/j.eurpsy.2025.1744

**Published:** 2025-08-26

**Authors:** D. Mezri, A. Gharbia, S. Hamzaoui, U. Ouali, A. Aissa, R. Jomli

**Affiliations:** 1 Avicenne, Razi hospital, Mannouba, Tunisia

## Abstract

**Introduction:**

Hospitalization in psychiatry often marks a critical point in the management of mental health disorders. For elderly patients, this can be a first-time experience and is often associated with complex psychological, social, or medical factors. Due to the age, psychiatric symptoms may become more pronounced because of comorbidities, cognitive decline and social challenges. Understanding the profile, medical history, presenting complaints, and associated conditions of elderly patients admitted for the first time to psychiatric care is essential to improve the comprehensive care.

**Objectives:**

The objective of this study is to analyze the profile of elderly patients hospitalized for the first time in the psychiatric department, focusing on their medical history, presenting complaints, diagnoses, and comorbidities.

**Methods:**

It’s a retrospective study. We reviewed the files of all patients aged over 65 years old who were hospitalized in the Avicenne Psychiatric Department at Razi Hospital between September 2022 and September 2024.

**Results:**

We identified 22 patients with 16 men and 6 women. The average age of our patients was 68 years (ranging from 64 to 80 years). The majority had a secondary school education (47%), came from an urban background (81%) , were retired (54%), with a high socioeconomic status (42%), married (61%), parent of an average of 2 children and with a family history of mental disease (52%). A history of somatic illness was found in 61% of the patients. The reason for hospitalization was behavioral disorder in 73%, suicidal thoughts in 18.2% and refusal to eat in 8.8%. A history of psychiatric consultation without the need for hospitalization was found in 59% of the cases around the age of 51 years. In 18% of the cases, the onset of the disorder was acute, while it was progressive in the rest. Concerning the diagnosis, we observed mental confusion caused by an organic pathology in 9%, a purely neurological cause of the disorder in 14% ( dementia in 10% and Parkinson’s disease in 4%), a depressive episode in 31%, a manic episode within the context of bipolar disorder in 37%, and schizophrenia in 9%. Comorbidity with paranoid personality disorder was observed in 4 patients, all of them were females. For patients with a psychiatric diagnosis, a neurological comorbidity was found in 31.25% (25% dementia, 6.25% Parkinson’s disease).

**Conclusions:**

This study shows that elderly patients in their first psychiatric hospitalization have complex profiles affected by age-related factors and comorbidities. Diagnosis varied from mood to psychotic disorders, often with neurological issues. The frequent history of psychiatric consultations without hospitalization suggests that early intervention could help prevent more severe admissions. Understanding these profiles is crucial for improving care and treatment for elderly patients.

**Disclosure of Interest:**

None Declared

